# Unilateral biportal endoscopic lumbar interbody fusion: Radiographic and clinical outcomes in grade I lumbar spondylolisthesis

**DOI:** 10.1097/MD.0000000000049539

**Published:** 2026-07-03

**Authors:** Lihua Hu, Chongkai Gong, Yi Mao, Rui Guo, Shu Jiang, Junchao Zhang

**Affiliations:** a Department of Orthopaedics, The Quzhou Affiliated Hospital of Wenzhou Medical University, Quzhou People’s Hospital, Quzhou, China.

**Keywords:** lumbar spondylolisthesis, minimally invasive surgery, transforaminal lumbar fusion, unilateral biportal endoscopy

## Abstract

To evaluate the radiographic and clinical outcomes of unilateral biportal endoscopic lumbar interbody fusion (ULIF) in patients with grade I lumbar spondylolisthesis, 30 patients underwent ULIF between January 2023 and January 2024. Clinical and radiographic outcomes were analyzed, including operative time, intraoperative blood loss, hospital stay, fusion rate, and laboratory indices (Hb, CRP, CPK). All procedures were successfully completed without intraoperative complications. The mean operative time was 199.7 ± 36.5 minutes, blood loss 60.7 ± 29.6 mL, and hospital stay 9.2 ± 3.7 days. Low back pain Visual Analogue Scale decreased from 6.63 ± 1.00 to 0.83 ± 0.80, leg pain Visual Analogue Scale from 6.47 ± 1.41 to 0.63 ± 0.67, and Oswestry Disability Index from 58.47 ± 12.86 to 18.87 ± 7.67 at 12 months. The fusion rate at final follow-up was 100%. Laboratory findings indicated a transient decrease in Hb and increases in CRP and CPK on POD1, with recovery by POD3. ULIF is a promising short-term option for grade I lumbar spondylolisthesis, demonstrating effective reduction, spinal decompression, minimal soft tissue injury, and high short-term fusion success.

## 1. Introduction

Lumbar spondylolisthesis, defined as displacement between adjacent vertebral bodies, can cause spinal instability, lower back pain, and leg symptoms.^[1,2]^ Lumbar interbody fusion remains a standard surgical intervention to achieve vertebral reduction and decompression.^[3,4]^

Traditional open TLIF requires extensive muscle dissection, resulting in prolonged recovery and higher complication rates.^[5-7]^ Minimally invasive TLIF reduces tissue trauma but has a narrow operative corridor, challenging screw placement.^[8,9]^

Unilateral biportal endoscopic (UBE) technology, introduced by Heo et al,^[10]^ uses separate visualization and instrument channels to combine an expanded surgical field with minimal muscle disruption, facilitating lumbar fusion and decompression with faster recovery.^[11,12]^ This study aims to evaluate the radiographic and clinical outcomes of ULIF in patients with grade I lumbar spondylolisthesis.

## 2. Materials and methods

### 2.1. Patients

Inclusion criteria were: single-level degenerative spondylolisthesis with concordant imaging findings and clinical symptoms; failure of standardized conservative treatment over ≥3 months; and Meyerding grade I^[13]^ spondylolisthesis.

Exclusion criteria comprised: prior lumbar spine surgery; Meyerding grade II–IV^[13]^ spondylolisthesis; and declined informed consent.

### 2.2. Surgical techniques of ULIF

Following the induction of general anesthesia, the patient was positioned prone. The target spinal segment was localized using C-arm fluoroscopy, and the percutaneous entry points for pedicle screw placement were marked accordingly. After standard sterile draping, 4 pedicle guidewires were inserted under C-arm fluoroscopic guidance. The protruding ends of these wires were secured to cranial and caudal drapes using sterile clamps, and a sterile adhesive drape was applied.

A double incision was then made unilaterally. Through these incisions, sequential dilators created both an endoscopic viewing portal and an instrument working portal. The endoscope was introduced into the viewing portal. Via the working portal, soft tissue was cleared and hemostasis achieved using electrocautery. This allowed visualization of the facet joint and lamina. The inferior aspect of the superior lamina and the superior aspect of the inferior lamina were then resected, along with a partial facetectomy of the superior level. The ligamentum flavum was subsequently dissected and excised, exposing the underlying dura mater and nerve root. With the nerve root gently retracted, the posterior aspect of the intervertebral disc was adequately exposed. The annulus fibrosus was incised and the nucleus pulposus removed. After curettage of the cartilaginous endplates and placement of local bone graft into the disc space, an interbody fusion cage was inserted under fluoroscopic control. Adequate dural sac and nerve root mobility were confirmed. Having ensured hemostasis, the endoscopic portion of the procedure was concluded.^[14,15]^

Subsequently, the pedicle tracts were tapped over the guidewires, and pedicle screws were inserted. Correct screw placement was verified fluoroscopically. Finally, the longitudinal connecting rod was secured, the surgical site was irrigated, a drain was placed, and the incisions were closed.

### 2.3. Postoperative management and observation index

Postoperative management: patients received routine prophylactic antibiotics for 24 hours postoperatively. Surgical drains were removed on postoperative day (POD) 1 to 2 when output was <50 mL/24 h. Mobilization with lumbar bracing was initiated following drain removal. Repeat lumbar radiographs, CT, and MRI were obtained on POD 3. Follow-up radiographic evaluations were conducted at 3, 6, and 12 months.

Perioperative data collection: parameters included operative duration, intraoperative blood loss, wound healing status, and procedure-related complications.

Laboratory parameters: hemoglobin (Hb), c-reactive protein (CRP), erythrocyte sedimentation rate, and creatine phosphokinase (CPK) levels were measured preoperatively and on POD 1 and 3.

Radiographic evaluation: lumbar lordosis (LL); Segmental angle (SA); Slip percentage (SP); Spinal canal area (SCA); Disk height (DH); Fusion status was assessed using the Bridwell grading system.^[16]^

Clinical outcome measures: pain severity was evaluated with the Visual Analogue Scale (VAS), while functional recovery was measured using the Oswestry Disability Index (ODI).

### 2.4. Statistical analysis

Statistical analyses were performed using SPSS software (version 25.0; IBM Corp.). Continuous data are expressed as the mean ± standard deviation ($\$\bar{x}\pm s\$$). Normally distributed data were compared using 1-way analysis of variance (ANOVA), with post hoc pairwise comparisons conducted via the Student-Newman–Keuls method when applicable. For non-normally distributed data, the Kruskal-Wallis test was employed. A *P*-value < .05 was considered statistically significant.

## 3. Results

### 3.1. Demographic results

**Figure 1.**
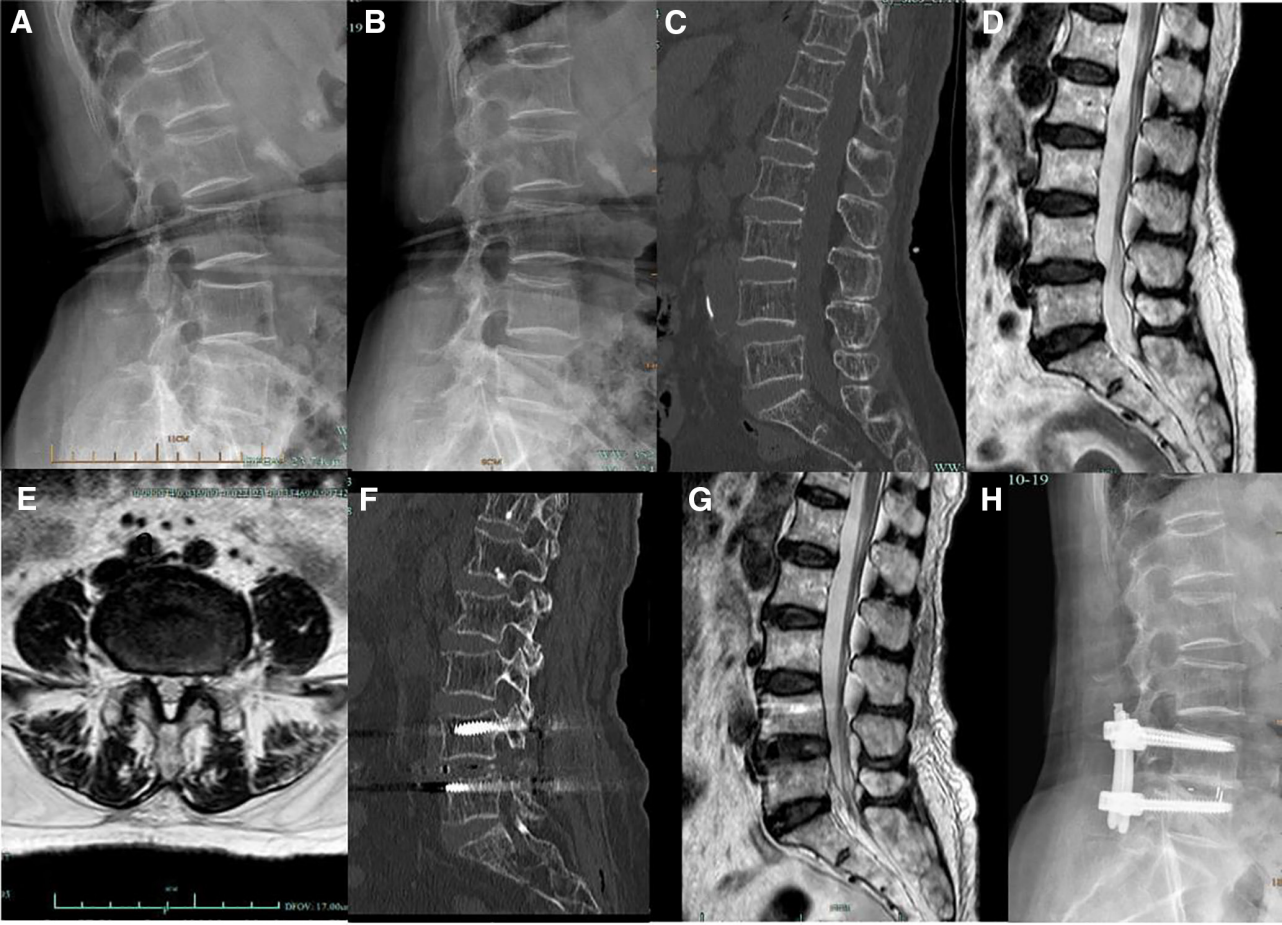
A 76-year-old female diagnosed with L4/5 spondylolisthesis and spinal stenosis, symptomatic for intermittent claudication after 100 meters of walking, was treated with L4/5 ULIF. Preoperative imaging revealed lumbar instability on flexion-extension radiographs (A, B) and L4/5 spondylolisthesis with spinal stenosis on CT (C) and MRI (D, E) scans. Postoperative CT (F) and MRI (G) revealed notable improvement, with a clear reduction in the L4/5 spondylolisthesis and significant alleviation of spinal stenosis. The 6-month follow-up radiograph (H) further indicated a successful outcome, demonstrating solid bony fusion at the L4/5 segment and stable fixation with no signs of screw loosening or migration. CT = computed tomography, ULIF = unilateral biportal endoscopic lumbar interbody fusion.

This study enrolled 30 patients (16 male, 14 female), with a mean age of 65.37 ± 8.50 years (range: 45–83 years; Table 1). Patient manifestations included lumbar disc herniation (n = 22), unilateral lower limb pain and numbness (n = 18), bilateral lower limb pain and numbness (n = 7), and isolated low back pain exacerbated by walking (n = 5). All participants underwent comprehensive imaging evaluations – including lumbar radiographs, dynamic flexion-extension films, CT, and MRI – to identify the primary pathological level responsible for symptoms. Affected levels comprised L3–L4 (n = 3), L4–L5 (n = 21), and L5–S1 (n = 6). A representative case was illustrated in Figure 1.

**Table 1:** Overall condition of the patient.

Category	Characteristic
Gender, n(%)	Male 16(53.33%) Female 14(46.67%)
Age/yr, ($\$\bar{x}\pm s\$$)	65.37 ± 8.50
Lesion segment, n(%)	L3/4, 3(10%) L4/5, 21(70%) L5/S1, 6(20%)
Clinical manifestation	Bilateral symptoms, 21(70%) Unilateral symptoms, 3(10%) Simple lower back pain, 6(20%)
Operation time/min, ($\$\bar{x}\pm s\$$)	199.67 ± 36.48
Blood loss in operation/mL, ($\$\bar{x}\pm s\$$)	60.67 ± 29.62
Hospital stay/d, ($\$\bar{x}\pm s\$$)	9.17 ± 3.67
Postoperative complications, n(%)	Cerebrospinal fluid leakage, 1(3.33%)

### 3.2. Clinical results

**Figure 2.**
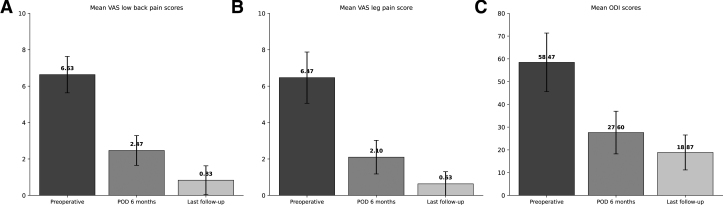
Comparison of VAS Back leg, VAS leg, and ODI score before and after operation. ODI = oswestry disability index, VAS = visual analogue scale.

All 30 patients successfully completed the surgery. The mean operative duration was 199.7 ± 36.5 minutes, with an average intraoperative blood loss of 60.7 ± 29.6 mL. The mean hospital stay was 9.2 ± 3.7 days. All incisions achieved primary healing without complications such as surgical site infection or venous thrombosis. One patient developed a cerebrospinal fluid leak, which resolved with conservative management, permitting discharge by postoperative week 2. During the minimum 12-month follow-up period, both the 6-month and final assessments demonstrated statistically significant reductions in low back pain (VAS), leg pain (VAS), and ODI scores compared to preoperative baselines (*P* < .05 for all outcomes; Fig. 2).

### 3.3. Clinical laboratory indexes

The laboratory test results of the patients are presented in Table 2. Compared with preoperative values, Hb levels showed a significant decrease on postoperative day 1 (*P* < .05), while CRP, erythrocyte sedimentation rate, and CPK levels exhibited marked elevations (*P* < .05). Notably, all parameters returned to normal ranges by postoperative day 3.

**Table 2:** Comparison of perioperative data ($\$\bar{x}\pm \;s\$$).

Index	Preoperative	POD 1 day	POD 3 day	*P*
HB (g/L)	134.97 ± 14.93	118.03 ± 14.76	124.83 ± 12.57	.001
ESR (mg/L)	12.03 ± 7.85	16.13 ± 7.89	12,90 ± 6.70	.09
CRP (mm/h)	1.49 ± 1.51	14.27 ± 8.51	7.19 ± 4.86	.002
CPK (U/L)	19.41 ± 7.45	34.01 ± 16.31	21.43 ± 8.18	.003

CPK = creatine phosphokinase, CRP = c-reactive protein, ESR = erythrocyte sedimentation rate, HB=hemoglobin, POD=postoperative day.

### 3.4. Radiological results

The radiographic outcomes are summarized in Table 3. Radiographic assessments were performed by 2 independent spine surgeons blinded to clinical outcomes, with disagreements resolved by consensus. Compared with preoperative measurements, significant improvements were observed at the 3-month follow-up, including increases in LL, SA, SCA, and DH (all *P* < .05), as well as reductions in SP. At the 3-month follow-up, 93.3% of patients achieved complete fusion according to the Bridwell grading system, with the remaining 2 cases attaining fusion by the 6-month follow-up. No instances of implant loosening, cage migration, or loss of reduction were observed during the follow-up period.

**Table 3:** Comparison of imaging results before and after surgery ($\$\bar{x}\pm \;s\$$).

Parameters	Preoperative	Postoperative 3 mo	Postoperative 6 mo	*P*
LL (°,$\$\;\;\;\bar{x}\pm s\$$)	38.47 ± 6.63	43.09 ± 4.88	42.13 ± 4.49	.002
SA (°,$\$\;\;\;\bar{x}\pm s\$$)	13.42 ± 2.64	19.26 ± 3.39	18.56 ± 3.31	.001
SP (%,$\$\;\;\;\bar{x}\pm s\$$)	17.61 ± 3.41	11.58 ± 4.08	12.23 ± 4.17	.003
SCA (cm^2^,$\$\;\;\;\bar{x}\pm s\$$)	58.90 ± 17.08	107 ± 23.07	101 ± 22.02	.001
DH (mm,$\$\;\;\;\bar{x}\pm s\$$)	9.29 ± 1.63	12.77 ± 1.63	12.00 ± 1.61	.002
Bridwell grading		21/7/2/0	28/2/0/0	.004

DH = disk height, LL = lumbar lordosis, SA = segmental angle, SCA = spinal canal area, SP = slip percentage.

## 4. Discussion

Lumbar interbody fusion is an effective treatment for lumbar spondylolisthesis, providing both spinal canal decompression and restoration of spinal stability.^[17,18]^ Traditional open surgery requires extensive detachment of the paraspinal muscles to expose the lamina, which can lead to significant damage to posterior spinal structures, often resulting in chronic postoperative back pain and muscle atrophy.^[19]^ With advances in endoscopic techniques, endoscopic lumbar fusion has gained widespread application.^[20]^ Heo et al^[10]^ were the first to combine UBE with TLIF, while Kim et al^[21]^ applied the ULIF technique to treat lumbar spondylolisthesis, both reporting favorable clinical outcomes. In this study, patients with grade I lumbar spondylolisthesis who underwent ULIF experienced significant improvements in low back pain, leg pain, and functional outcomes. Laboratory markers (CPK, CRP) indicated minimal soft tissue injury, supporting the minimally invasive nature of the procedure.^[22]^ The observed radiographic improvements, including increased LL, segmental angle, SCA, and disc height, demonstrate that ULIF provides effective reduction and decompression while maintaining sagittal alignment. The high short-term fusion rate observed is consistent with previous studies on ULIF.^[23,24]^ Compared to traditional open TLIF or minimally invasive TLIF, ULIF offers reduced blood loss, accelerated recovery, and minimal paravertebral muscle disruption.^[5-9,11,12]^ However, direct comparative studies between unilateral and biportal techniques are necessary to more accurately assess relative efficacy.

Imaging studies before and after surgery revealed notable increases in SCA and disc space height, as well as improved LL. Short-term follow-up indicated no screw loosening or loss of reduction, with solid bony fusion achieved in all cases. These findings collectively demonstrate that the ULIF technique allows for adequate decompression, minimizes soft tissue injury, helps restore spinopelvic alignment, and leads to satisfactory clinical outcomes in the treatment of lumbar spondylolisthesis.

This study included 30 patients with grade I lumbar spondylolisthesis who underwent ULIF surgery. The mean operative time was 199.67 ± 36.48 minutes, with an average intraoperative blood loss of 60.67 ± 29.62 mL. The mean hospital stay was 9.17 ± 3.67 days. Post-discharge follow-up revealed significant symptomatic improvement: the back VAS score decreased from 6.63 ± 1.00 preoperatively to 0.83 ± 0.80, the leg VAS score dropped from 6.47 ± 1.41 to 0.63 ± 0.67, and the ODI score improved from 58.47 ± 12.86 to 18.87 ± 7.67. These outcomes are consistent with those reported by He et al,^[23]^ who conducted a retrospective analysis of 28 patients treated with ULIF. In their cohort, the mean operative time was 162.2 ± 14.3 minutes, and the average blood loss was 149.6 ± 7.3 mL. Postoperative VAS scores decreased from 6.3 ± 0.8 to 1.2 ± 0.7, while ODI scores improved from 46.0 ± 3.1 to 19.3 ± 1.7, demonstrating clinical efficacy comparable to that observed in the present study.

Serum CRP and CPK levels, which are established biomarkers for surgical trauma and muscle injury, exhibited a marked increase on the first postoperative day, followed by a rapid decline by the third day. This swift reduction may be attributed to the minimally invasive nature of ULIF. ULIF minimizes extensive dissection of the paravertebral muscles by utilizing a separate working channel to access the surgical site.^[25,26]^ This approach provides a stable operative field while reducing the need for sustained tissue retraction. Furthermore, continuous irrigation during the surgery may help remove local inflammatory mediators, thereby potentially mitigating the systemic inflammatory response. The rapid return of CPK levels to normal was particularly indicative of the limited muscle tissue damage associated with the ULIF technique.^[27]^

SP was a reliable parameter for evaluating reduction. Postoperative SP showed substantial improvement compared to preoperative values. Moreover, although a slight decrease in SP was observed at the 3-month follow-up compared to immediate postoperative measurements, the difference was not statistically significant, indicating that the procedure achieved and maintained stable fixation. SCA and DH were direct determinants of spinal canal volume and the degree of nerve root decompression. Following ULIF surgery, significant increases in both SCA and DH were observed, indicating adequate intraoperative decompression and nerve root release.^[24]^ Consequently, this led to a substantial reduction in lower limb pain scores. These findings were supported by a study by Zhu et al,^[28]^ which analyzed 28 patients with degenerative spondylolisthesis undergoing ULIF. Their results demonstrated a marked increase in SCA (from 69.70 ± 14.66 mm^2^ to 175.83 ± 58.76 mm^2^) and DH (from 7.12 ± 1.37 mm to 10.47 ± 0.69 mm), alongside a reduction in SP from 11.07 ± 3.70% to 3.67 ± 1.45%.^[28]^ The study confirms that ULIF effectively achieves reduction, adequately decompresses the nerve roots, and results in significant postoperative relief of both lower back and lower limb pain.

LL and SA were critical parameters for maintaining sagittal spinal balance. Inadequate restoration of LL could lead to postoperative low back pain and long-term functional impairment, while insufficient correction of SA may compromise segmental stability.^[29,30]^ A follow-up study demonstrated that ULIF effectively improved these angles, with LL increasing from 45.5 ± 1.1° to 48.0 ± 1.5° and SA from 7.9 ± 0.8° to 9.4 ± 0.7°.^[23]^ In the present study, both LL and SA showed significant improvement compared to preoperative values. This improvement indicates that the ULIF procedure can successfully reconstruct sagittal spinal balance, thereby ensuring favorable long-term outcomes.

The fusion rate was a critical indicator for assessing the success of lumbar fusion surgery. In patients with spondylolisthesis, early fusion reduces the risk of postoperative slip progression, while a high fusion rate is essential for sustaining long-term outcomes.^[31]^ In the present study, a fusion rate of 93.3% was achieved at 3 months, with all patients achieving solid bony fusion by 6 months. This favorable outcome may be attributed to the ULIF technique, which allows for thorough removal of the cartilaginous endplate under direct endoscopic visualization while minimizing structural damage, thereby enhancing the environment for fusion. Furthermore, compared with previous studies,^[27,32]^ the greater postoperative DH observed here indicates that a larger interbody cage could be implanted, facilitating substantial bone grafting and further supporting the high fusion rate. Additionally, the lumbar spine bears higher mechanical loads than thoracic and cervical segments,^[33]^ which underscores the importance of achieving stable reduction and fusion.

This study has several limitations. It is retrospective, has a small sample size, lacks a direct comparison group, and radiographic follow-up was limited to 6 months, while clinical follow-up extended to 12 months. Long-term fusion stability, cage subsidence, and adjacent segment degeneration require extended observation in future studies.

## 5. Conclusion

Unilateral biportal endoscopic lumbar interbody fusion (ULIF) is a promising short-term surgical option for patients with grade I lumbar spondylolisthesis, demonstrating effective reduction, sufficient spinal canal decompression, minimal soft tissue injury, and high short-term fusion rates. Given the preliminary nature of this study and the lack of a direct comparison group, further prospective, comparative studies are warranted to confirm these findings.

## Author contributions

**Conceptualization:** Junchao Zhang.

**Data curation:** Chongkai Gong.

**Formal analysis:** Chongkai Gong, Rui Guo.

**Funding acquisition:** Lihua Hu.

**Investigation:** Lihua Hu, Yi Mao.

**Validation:** Yi Mao.

**Writing – original draft:** Lihua Hu, Chongkai Gong.

**Writing – review & editing:** Rui Guo, Shu Jiang, Junchao Zhang.
